# Bacterial community analysis on the different mucosal immune inductive sites of gastrointestinal tract in Bactrian camels

**DOI:** 10.1371/journal.pone.0239987

**Published:** 2020-10-08

**Authors:** Wang-Dong Zhang, Wan-Ling Yao, Wan-Hong He, Jian-Fei Li, Xiu-Ping Wu, Zhi-Hua Chen, Lei Liu, Wen-Hui Wang

**Affiliations:** College of Veterinary Medicine, Gansu Agricultural University, Lanzhou, Gansu, China; University of Illinois, UNITED STATES

## Abstract

The microbial communities colonize the mucosal immune inductive sites could be captured by hosts, which could initiate the mucosal immune responses. The aggregated lymphoid nodule area (ALNA) and the ileal Payer’s patches (PPs) in Bactrian camels are both the mucosal immune inductive sites of the gastrointestinal tract. Here, the bacteria community associated with the ALNA and ileal PPs were analyzed using of 16S rDNA-Illumina Miseq sequencing. The mutual dominant bacterial phyla at the two sites were the Bacteroidetes, Firmicutes, Verrucomicrobia and Proteobacteria, and the mutual dominant genus in both sits was *Prevotella*. The abundances of the Fibrobacter, Campylobacter and RFP12 were all higher in ALNA than in ileal PPs. While, the abundances of the 5-7N15, Clostridium, and Escherichia were all higher in ileal PPs than in ALNA. The results suggested that the host’s intestinal microenvironment is selective for the symbiotic bacteria colonizing the corresponding sites, on the contrary, the symbiotic bacteria could impact on the physiological functions of this local site. In ALNA and ileal PPs of Bactrian camel, the bacteria which colonized different immune inductive sites have the potential to stimulate different immune responses, which is the result of the mutual selection and adaptation between microbial communities and their host.

## Introduction

Commensal microbiota densely colonize the mammalian gastrointestinal mucosal surface. They can even reach 10^12^ cells/g and the number of species is up to 500–1000 in colon contents [1–3], and their encoding genes are more than their hosts’ genome [4]. These complex microbial communities play an important role in host’s immune system. For instance, commensal *segmented filamentous bacteria* could promote the development of Peyer’s patches (PPs), stimulate the response of T helper 17 (Th17) cells and the production of T-dependent IgA [5] in small intestine. Moreover, some Clostridium could induce regulatory T (Treg) cells to exert the systemic anti-inflammatory effect [6, 7]. Furthermore, bacterial metabolites such as short chain fatty acids (SCFAs) can promote the differentiation of the B cells, enhance the production of host’s antibodies [8], and improve the proliferation and function of intestinal colonic Treg cells in special pathogen free (SPF) mice and sterile animals [9]. Also, some certain *Bacteroides* could promote the colonization of intraepithelial lymphocytes (IELs) and secretion of interleukin 6 (IL-6) [10]. Therefore, the intestinal flora and their metabolites not only provide necessary nutrition for the host and compete with pathogenic bacteria for the same colonization niche, but also play an important role in regulating adaptive and natural immune response of the host [11, 12].

Nodule-likePPs, an organized lymphoid tissue, mainly distributed in the ileal mucosa and submucosa, are the classic inductive site for gastrointestinal mucosal immunity to capture antigens, induce mucosal immune responses and regulate mucosal immune homeostasis [13, 14]. Interestingly, there is a large area of aggregated lymphoid nodule area (ALNA) in the abomasum of Bactrian camels, which was firstly reported by Wen-hui Wang [15, 16]. This triangle-like structure starts from ventriculi neck and back along the ventral wall of the curvature ventriculi minor and is composed of the reticular mucosal folds region (RMFR) and longitudinal mucosal folds region (LMFR) [15, 17]. Furthermore, ALNA, an organized lymphoid tissue, with the same histological characteristics as the ileal PPs, is a unique and important area of the mucosal immune inductive site in Bactrian camel abomasum [15, 18].

At present, the studies on the bacterial community in the gastrointestinal tract of camels mainly focused on rumen, intestines, and/or feces, and the results suggested that the composition of microbial community in the rumen of a camel was similar to other ruminants with differences in the abundance, meanwhile, there were different metagenomic profiles in different digestive tract regions [19, 20]. However, the studies about the different mucosal immune inductive sites of gastrointestinal tract has only received few attentions. Hence, based on our previous histological and structural studies of ALNA and ileal PPs in Bactrian camels [16, 18, 21–23], 16S rDNA-Illumina Miseq sequencing was used to comparatively analyze the bacteria community characteristics in ALNA and ileal PPs of Bactrian camels, moreover, the biological information hidden in them were revealed in this study.

## Materials and methods

### Ethics statement

All experimental procedures were approved by the Animal Care and Use Committee (IACUC) of College of Veterinary Medicine of Gansu Agricultural University (Approval No: GSAU-AEW-2016-0005). All efforts were made to minimize animal suffering.

### Experimental design and sample collection

All Bactrian camels were distributed in Minqin county, Gansu province, China. They mainly eat *Agriophyllum pungens*, *Ceratoides latens*, and *Nitraria tangutorum* etc. when grazing time. All the six healthy pubertal Alashan Bactrian camels (3 to 5 years old, 3 males and 3 females) were randomly selected, anaesthetised intravenously with sodium pentobarbital (20 mg/kg), and exsanguinated until death. After the abdominal cavity was opened, the whole abomasum (from cardia to pylorus) and ileum were cut, and their food residues were gently washed with sterilized saline, then the mucus was quickly scraped from the mucosal surface of ALNA (including RMFR and LMFR) and ileal PPs in each Bactrian camel under a sterile operation. A total of 18 samples were collected from the above three locations of six Bactrian camels, and quickly put into the 2.5 ml cryopreserved tube and kept in liquid nitrogen for further use.

### Extraction of genome DNA

The total genome DNA was extracted from samples using the TIANamp Stool DNA Kit (TIANGEN, China) according to manufacturer’s instructions. 1% agarose gels were used to detect the integrity and impurity in the extracted bacterial DNA samples. The purity and concentration were tested by NanoPhotometer spectrophotometer (IMPLEN, Munich, Germany) and Qubit 2.0 Flurometer (Life Technologies, Carlsbad, CA, USA), respectively. According to the concentration, DNA was diluted to 1 ng/μL using sterile water.

### PCR amplification and sequencing

The V3-V4 hypervariable regions of the bacteria 16S rRNA gene were amplified with primers 341F (5’-CCTACGGGNGGCWGCAG-3’) and 805R (5’-GACTACHVGGG-TATCTAATCC-3’) [24] on the GeneAmp® PCR System 9700 (Applied Biosystem, USA). All PCR reactions were carried out in 30 μL reactions with 15 μL of Phusion^®^ High-Fidelity PCR Master Mix (Biolabs, New England); 0.2 μM of forward and reverse primers, and about 10 ng of template DNA. Thermal cycling was consisted of initial denaturation at 95°C for 3 min, followed by 25 cycles of denaturation at 95°C for 30 s, annealing at 55°C for 30 s, and elongation at 72°C for 30 s, finally 72°C for 5 min. The same volume of 1× loading buffer (contained SYB green) was mixed with PCR products and electrophoresis on 2% agarose gel was operated for detection. Samples with bright main strip about 460 bp (V3-V4) were chosen for further experiments. PCR products were mixed in equidensity ratios. Then the mixture of PCR products were purified with GeneJET Gel Extraction Kit (Thermo Scientific, Waltham, MA, USA). Sequencing libraries were generated using NEB Next^®^ Ultra^TM^ DNA Library Prep Kit for Illumina (NEB, USA) following manufacturer’s recommendations and index codes were added. The library quality was assessed on the Qubit@ 2.0 Fluorometer (Life Technologies, CA, USA) and Agilent 2100 Bioanalyzer (Agilent Technologies, Palo Alto, CA). At last, the library was sequenced on an Illumina MiSeq platform and 250 bp paired-end reads were generated. All the sequencing data were deposited in the National Center for Biotechnology Information Sequence Read Archive (NCBI SRA) under accession no. PRJNA624714.

### Sequencing data processing and analysis

The raw data were filtered to obtain clean reads by removing adapter-polluted and low-quality (average quality lower than 19 with PHRED algorithm) reads and the reads with N bases > 5% etc. The Q30 percentages of the clean reads were all greater than 85% after filtering. According to overlap of the clean data, we spliced the paired reads using the PEAR **(**v0.9.10) software [25]. After merging, the sequences were removed chimeras and clustered into operational taxonomic units (OTUs) with UCLUST [26] based on a 97% pairwise identity. Subsequently, the RDP classifier (v2.2) was used to annotate the taxonomic information for each representative sequence against the Greengene database (201305) [27]. Alpha diversity indices, including Chao 1, Shannon, and Simpson were calculated with MOTHUR V1.31.2 software [28]. Rarefaction curves of Observed species were plotted using R (v3.1.1) software. Beta diversity analysis used to investigate the diversity between different groups was performed with QIIME (v1.80) [29]. The hierarchical clustering using Bray-Curtis distances based on relative abundance of species was done to cluster the data set. And the difference among the groups were analyzed by the principal coordinate analysis (PCoA) based on the distance matrices of weighted UniFrac. Species composition analysis including Venn diagrams and composition diagrams of the microbiota were performed using R (v3.1.1) software. Venn diagrams were generated to visualize the OTUs shared among the three groups. In addition, we plotted the community bar graphs which present what microorganisms in a taxonomy level and the relative abundance of each microbe in each sample. Kruskal–Wallis test analysis were used to determine if there were statistically significant differences among the alpha diversity indices data and the relative abundances data of microbial taxa using IBM SPSS v. 23.0. (SPSS, Chicago, USA). Statistical significance was accepted as p < 0.05 and extreme significance was accepted as p < 0.01, adjusted for ties. The phylogenetic tree analysis were completed with FASTTREE V2.1.3 [30]. Besides, in order to identify the major contributor bacteria in each group, LEfSe analysis was performed by online LEfSe software [31].

## Results

### Sequencing data and microbial alpha-diversity

**Fig 1:**
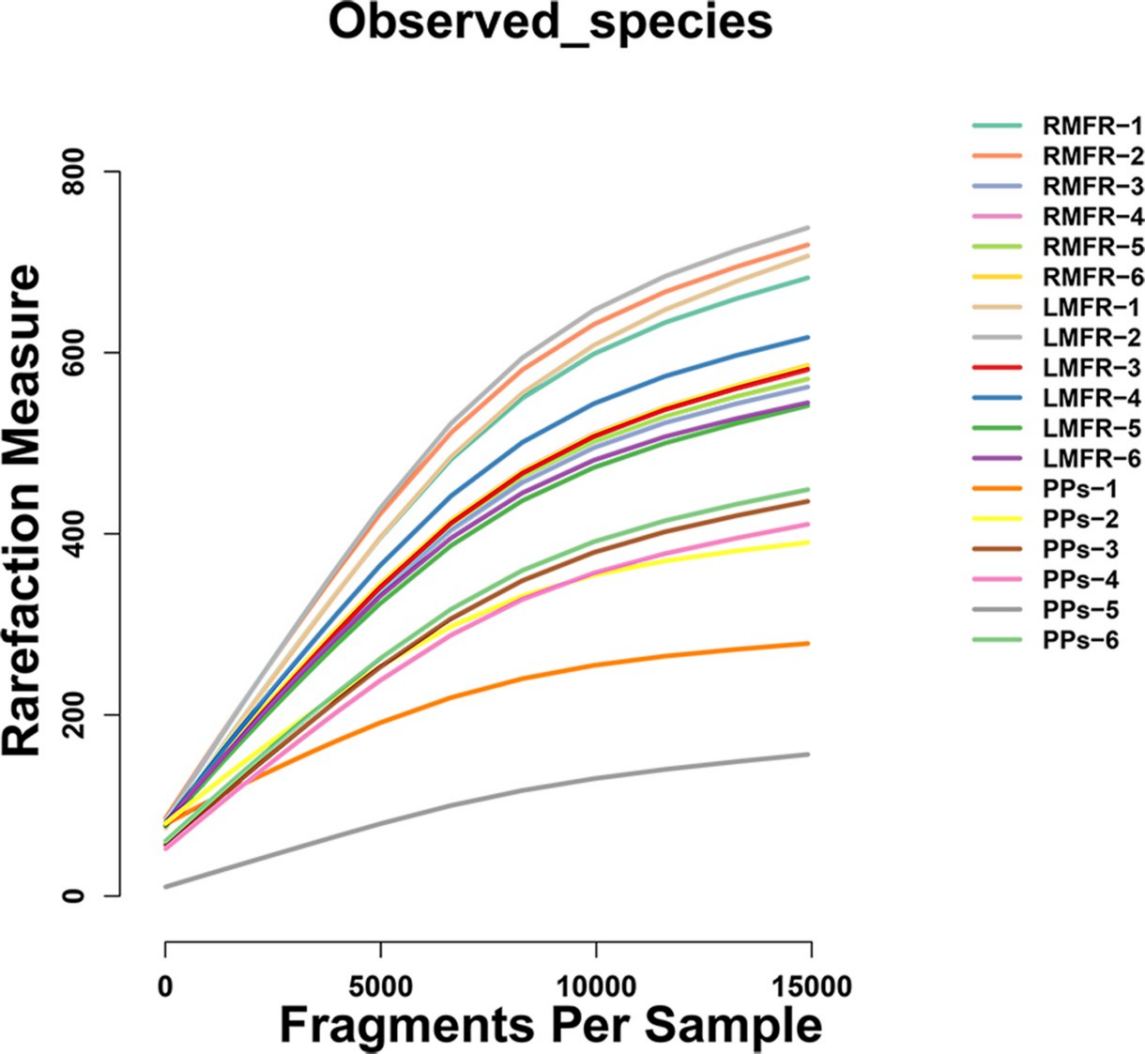
Rarefaction curves of each sample from LMFR, RMFR and ileal PPs of Bactrian camels calculated at the species level. Horizontal axis: the amount of effective sequencing data; vertical axis: the observed number of operational taxonomic units (OTUs). Total sample abundance estimates were calculated by abundance estimators Observed species. LMFR: longitudinal mucosal folds region; RMFR: reticular mucosal folds region; PPs: ileal Payer’s patches.

After the quality control, a total of 872,650 sequence reads were obtained from all the samples and grouped into 2,017 OTUs at the 97% similarity cut-off level. There was a similar pattern in the rarefaction curves: the number of OTUs of all samples gradually increased with the increase of the number of measured sequences, furthermore the curves became more gentle and the increasing trend became smaller, indicating that most of species existing in each sample were observed (Fig 1).

**Fig 2:**
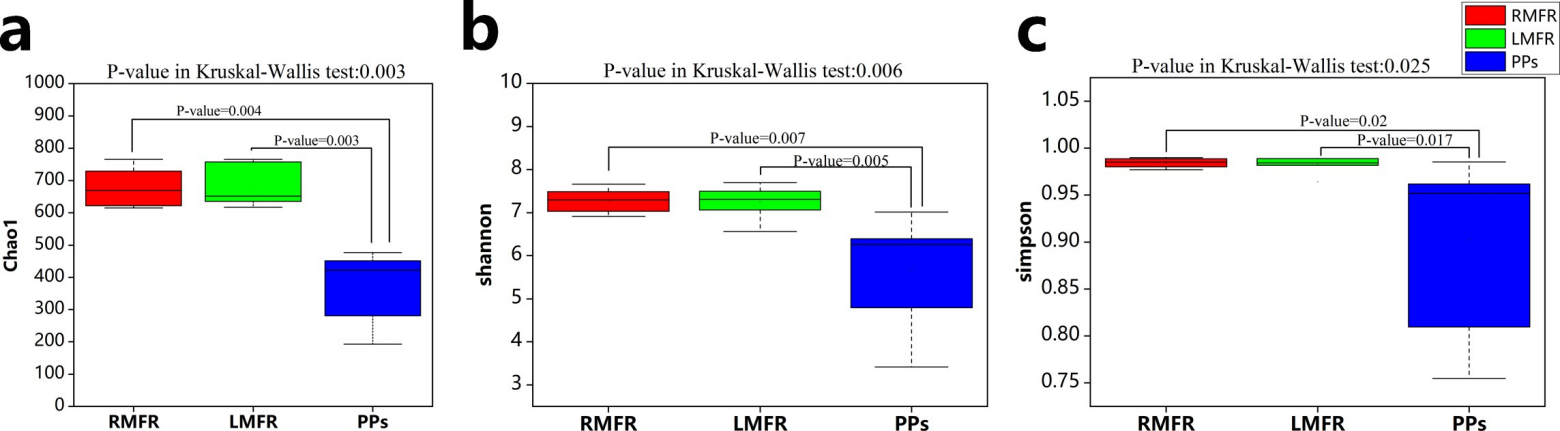
The alpha-diversity comparison among LMFR, RMFR and ileal PPs three sites. (a) Chao index, (b) Shannon index and (c) Simpson index. P<0.01 indicates extremely significant difference between two groups connected with lines, and P>0.05 indicates not significant difference. LMFR: longitudinal mucosal folds region; RMFR: reticular mucosal folds region; PPs: ileal Payer’s patches.

The alpha-diversity of the microbial communities in ALNA and ileal PPs of Bactrian camels was estimated by Chao, Shannon and Simpson indices which are listed in Tables 1 and S1. The results revealed that the Chao, Shannon and Simpson indices in LMFR and RMFR were all extremely higher than in ileal PPs (P<0.01), but there was no significant difference in Chao, Shannon and Simpson indices between LMFR and RMFR (P>0.05) in Fig 2.

**Table 1:** Number of clean sequence reads and OTUs and the values of alpha diversity indices in each group.

Sample	Nseqs	NOTUs	alpha diversity		
			chao1	shannon	simpson
RMFR	98502.67±6913.04	630.83±58.79	678.23±60.92	7.28±0.28	0.98±0.005
LMFR	96245.00±3965.22	631.50±68.01	679.92±64.70	7.24±0.40	0.98±0.009
PPs	96135.67±3329.56	358.83±106.09	374.35±112.90	5.69±1.33	0.90±0.095

Note: Nseqs indicates the number of sequence clean reads; NOTUs indicates the number of OTUs; the data are expressed as mean ± SD.

LMFR: longitudinal mucosal folds region; RMFR: reticular mucosal folds region; PPs: ileal Payer’s patches.

### OTUs distribution of the microbial community in the LMFR, RMFR and ileal PPs

**Fig 3:**
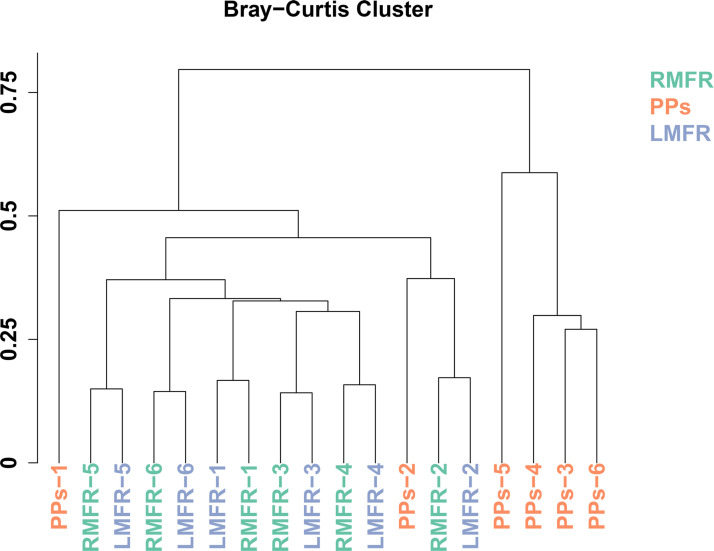
The hierarchical cluster analysis of Bray–Curtis distances generated from taxa tables summarized at the operational taxonomic unit (OTU) level. LMFR: longitudinal mucosal folds region; RMFR: reticular mucosal folds region; PPs: ileal Payer’s patches.

**Fig 4:**
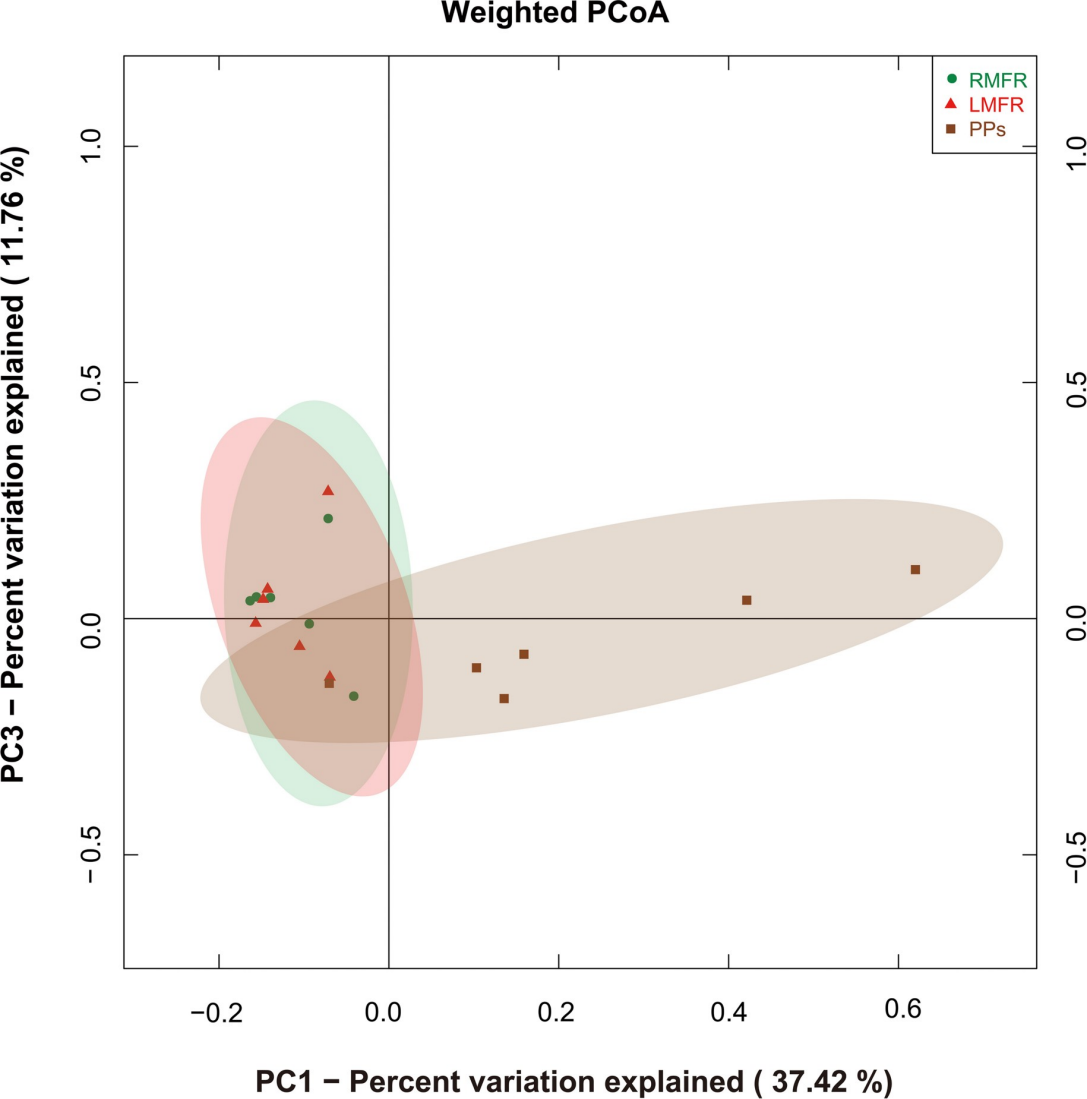
Weighted version of UniFrac-based principal coordinate analysis (PCoA) plot with PC1 and PC3 at the operational taxonomic unit (OTU) level among LMFR, RMFR and ileal PPs. LMFR: longitudinal mucosal folds region; RMFR: reticular mucosal folds region; PPs: ileal Payer’s patches.

In order to understand the similarities of microbial community in the LMFR, RMFR and ileal PPs of Bactrian camels, hierarchical clustering analysis was performed based on Bray-Curtis distances generated from the OTU abundance. The results showed that the all samples could be grouped into two clusters: ALNA (LMFR and RMFR) and ileal PPs clusters (Fig 3). Among the clusters, the grouped LMFR and RMFR samples indicated that they had similar bacteria compositions. In addition, we performed PCoA of weighted UniFrac distance based on the OTUs of the microbial communities. It can be seen from PCoA scatter plot that PPs group was obviously separated from LMFR and RMFR groups (Fig 4).

**Fig 5:**
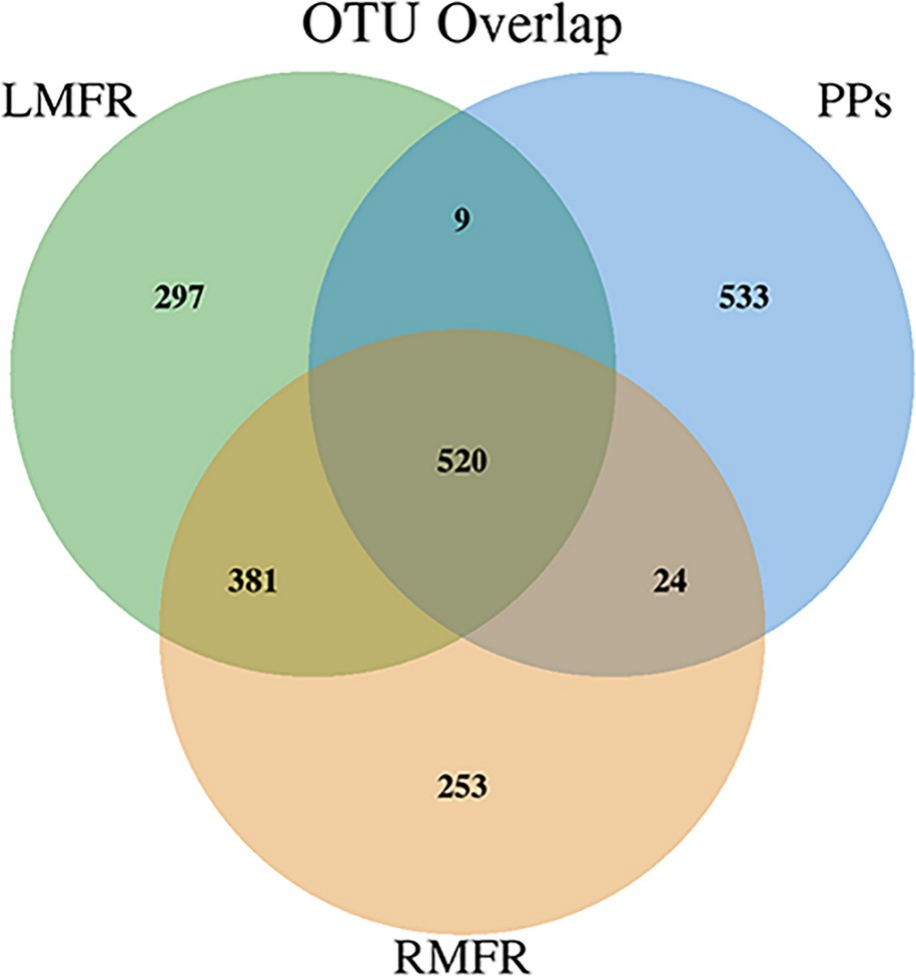
Venn profile of microbiota OTUs in the LMFR, RMFR and ileal PPs of Bactrian camels. LMFR: longitudinal mucosal folds region; RMFR: reticular mucosal folds region; PPs: ileal Payer’s patches.

Venn diagram was made according to the overlapping OTUs among the LMFR, RMFR and ileal PPs, then the mutual and unique OTU numbers were calculated in each group. The results showed that the OTU counts were 1207 in LMFR, 1178 in RMFR, and 1086 in ileal PPs; 520 OTUs were shared among the above three sites and accounted for 43.1%, 44.1% and 47.9%, respectively. Between LMFR and RMFR, there were 901 identical OTUs accounted for 74.6% and 76.5%, respectively. Between LMFR and PPs, there were 529 identical OTUs accounted for 43.8% and 48.7%, respectively. Besides, between RMFR and PPs, there were 544 identical OTUs accounted for 46.2% and 50%, respectively (Fig 5). We can conclude that the number of identical OTUs between LMFR and RMFR were more than between LMFR and PPs and between RMFR and PPs.

### Microbial composition in the LMFR, RMFR and ileal PPs at phylum and genus levels

**Fig 6:**
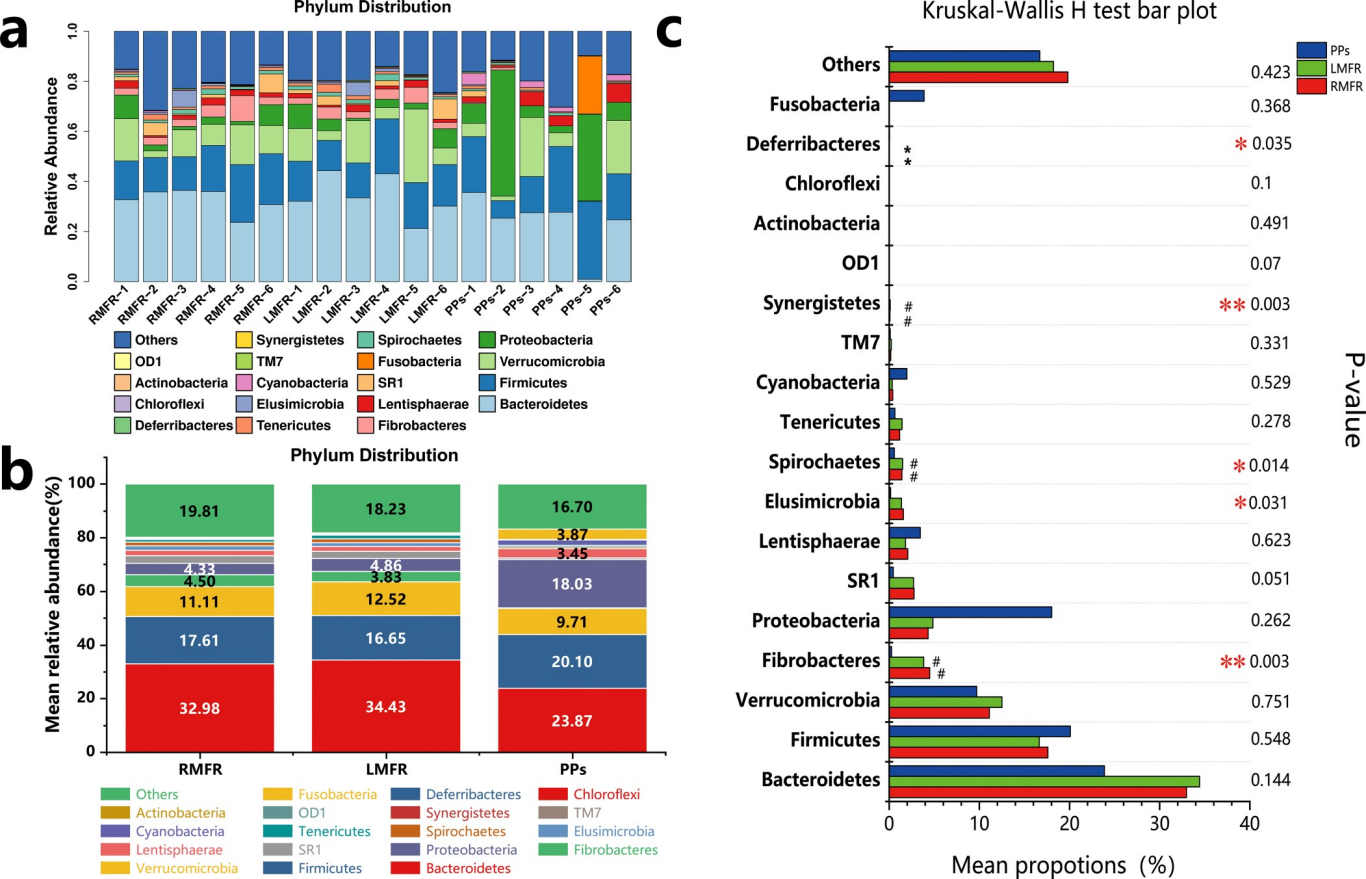
The relative abundance of phylotypes at the phylum in the samples from LMFR, RMFR and ileal PPs. (a) Composition and relative abundance of microbes at the phylum level for each sample. (b) Composition and mean relative abundance of microbes at the phylum level for each site. (c). Kruskal-Wallis test for differences in these microbial taxa among three sites at the phylum level. LMFR: longitudinal mucosal folds region; RMFR: reticular mucosal folds region; PPs: ileal Payer’s patches.

All sequences were identified and subjected to different taxonomic levels. The results showed that 14 phyla and 46 genera in RMFR, 14 phyla and 41 genera in LMFR, and 16 phyla and 59 genera in PPs were detected (S2 and S3 Tables). At the phylum level, the most prevalent 12 phyla in all samples were Bacteroidetes, Firmicutes, Verrucomicrobia, Proteobacteria, Lentisphaerae, Fibrobacteriae, SR1, EluSimicrobia, Spirochaetes, Tenericutes, Cyanobacteria and TM7 contributing smaller proportions of the sequencing reads. The rest of phyla were also presented but at lower abundance rates (Fig 6A). Then Kruskal–Wallis tests were performed on the phyla-level taxa, 5 phyla have a significant difference in abundance in at least one group (FDR-corrected Kruskal–Wallis test: p < 0.05) (Fig 6C). There were no significant differences among the levels of Bacteroidetes, Firmicutes, Verrucomicrobia, Proteobacteria, SR1, Lentisphaerae, Tenericutes, Cyanobacteria and TM7. Bacteroidetes, Firmicutes, Verrucomicrobia and Proteobacteria were the mutual predominant phyla of RMFR, LMFR and ileal PPs and accounted for 66.03% in RMFR, 68.46% in LMFR, 71.62% in ileal PPs respectively (Fig 6B). The abundance of Fibrobacteres, Elusimicrobia and Spirochaetes in ALNA (both RMFR and LMFR) were significantly higher than in ileal PPs. Unexpectedly, the abundance of Fusobacteria was higher in ileal PPs.

**Fig 7:**
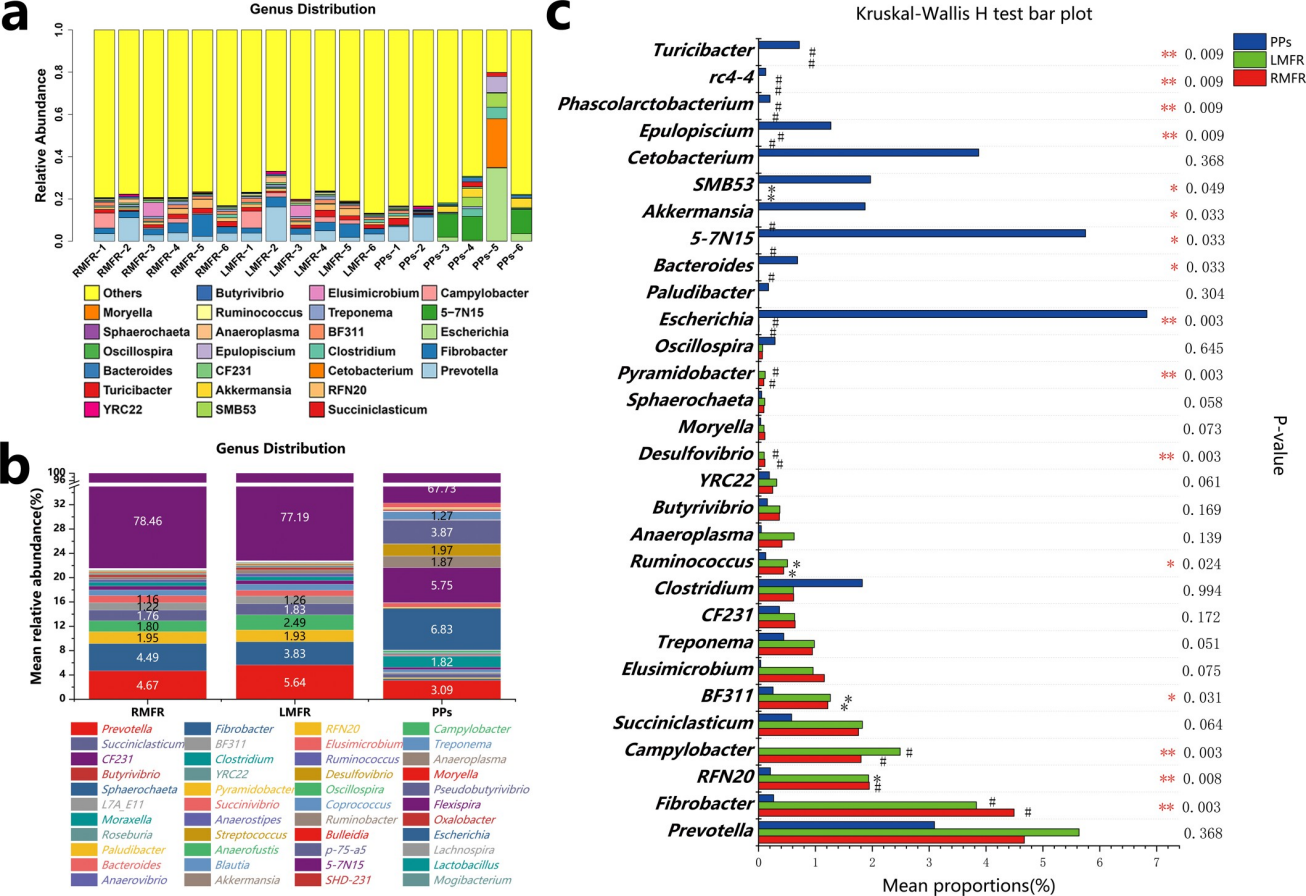
The relative abundance of phylotypes at the genus level in the samples from LMFR, RMFR and ileal PPs. (a) Composition and relative abundance of microbes at the genus level for each sample. (b) Composition and mean relative abundance of microbes at the genus level for each site. (c). Kruskal-Wallis test for differences in these microbial taxa among three sites at the genus level. LMFR: longitudinal mucosal folds region; RMFR: reticular mucosal folds region; PPs: ileal Payer’s patches.

**Fig 8:**
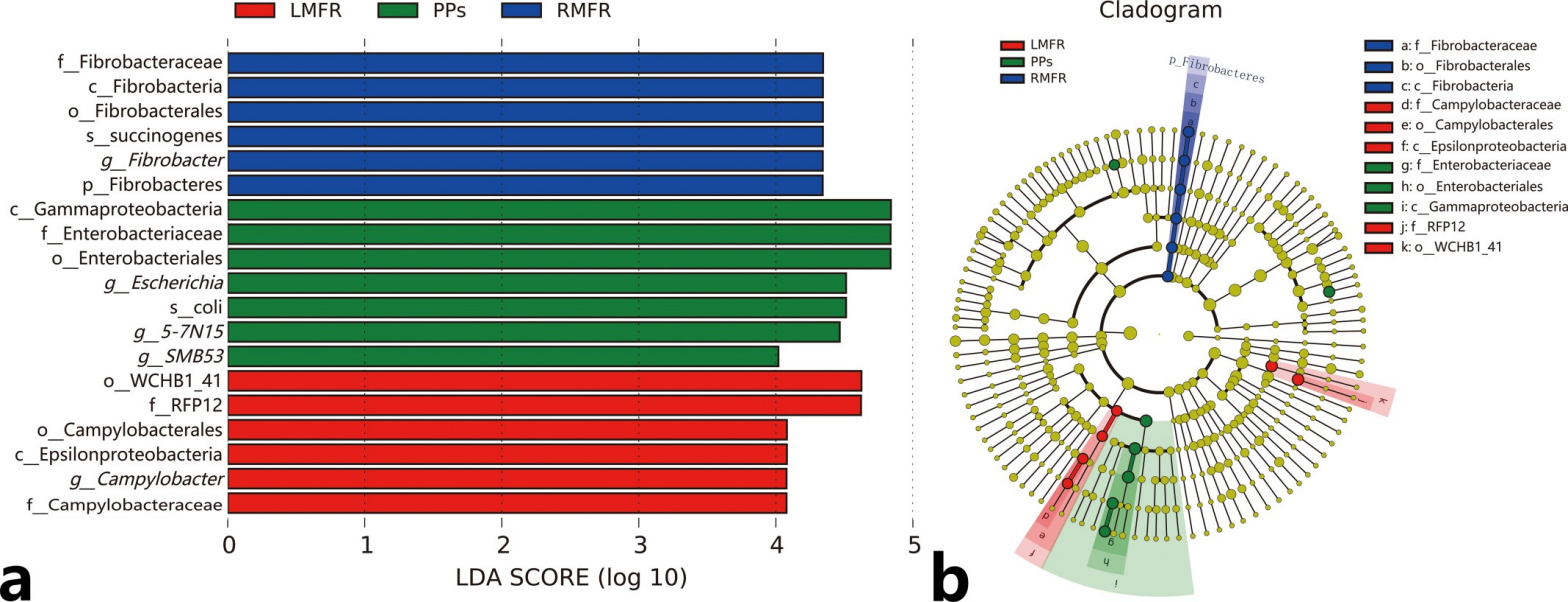
Different mucosal inductive sites and line-specific biomarkers. (a) LDA score distribution histogram. LDA score > 4.0 indicates differentially abundant genera which was considered as biomarkers. (b) LEfSe cladogram. The root of the cladogram denotes the domain bacteria. Different colors indicating the different mucosal inductive sites hosting the greatest abundance. The size of each node represents their relative abundance. LMFR: longitudinal mucosal folds region; RMFR: reticular mucosal folds region; PPs: ileal Payer’s patches.

To further characterize taxonomic differences, the most prevalent 25 genera were characterized (Fig 7A), then we performed Kruskal–Wallis tests on genus-level taxa, (Fig 7B and 7C). The abundance of *Prevotella* (accounted for 4.67% in the RMFR, 5.64% in the LMFR, 3.09% in the ileal PPs) was the highest, and there were no differences among the three sites. Sixteen genera showed significant differences in abundances among the three sites: the abundances of *Fibrobacter*, *RFN20*, *Campylobacter*, *BF311*, *Ruminococcus*, *Desulfovibrio* and *Pyramidobacter* in the RMFR and LMFR were significantly higher than in ileal PPs, but the abundances of *Escherichia*, *Bacteroides*, *5-7N15*, *Akkermansia*, *Epulopiscium*, *Phascolarctobacterium*, *rc4-4* and *Turicibacter* in ileal PPs were higher than in RMFR and LMFR (Fig 7B), Moreover, the LEfSe analysis results showed that Fibrobacteres, *Campylobacter* and *RFP12* were the main contributing bacteria in ALNA, while *5-7N15*, *Clostridium*, and *Escherichia* were the main contributing bacteria in ileal PPs (Fig 8).

## Discussion

### The relationship between the host and common dominant bacteria

The PPs were mainly distributed in the terminal ileum. They played an important role in the regulation of the digestive tract mucosal immune homeostasis through capturing the antigen and mainly inducing the IgA production. ALNA, a special organized lymphoid tissue, was discovered in the abomasum of Bactrian camels [16]. The structure of ALNA is highly similar to ileal PPs. In this study, the common dominant bacteria colonized the mucosa surface of the ALNA and ileal PPs were the same as in the intestine of other animals, such as dromedary [20], Bactrian camel [19], cattle [32], sheep [33] and horse [34]. There are several reasons for this phenomenon: first, the similar microenvironment of the intestine, such as the same temperature (maintained at about 37–40°C) in vivo [35], which provided the foundation for the stable reproduction of microorganisms; second, the similar chyme components, for the herbivores, including large amounts of plant cellulose, fructose, starch granules, etc.[3, 36], which could provide similar nutrients for the microorganisms; third, similar strategies of the immune defense, for instance in different herbivores, the classical cell for capturing antigens was the microfolds cell (M cell), and the dominant immunoglobulin (Ig) located in the lumen was SIgA [37], rather than other Ig molecules. Conversely, if these factors significantly changed, the intestinal flora could dramatically change accordingly. Therefore, these common predominant bacteria had been activated and selected by the host’s internal environment, chyme and immunity together.

Accordingly, these common predominant bacteria also played a crucial role in the host’s nutrient absorption and immune defense, such as synthesizing glycoside hydrolase and polysaccharide lyase, promoting the degradation of the fibrosome, fructose, starch, etc., and providing the five-carbon or six-carbon sugars [36, 38]; the bacterial components (such as LPS [39]) and metabolites (such as SCFAs [40, 41], antimicrobial peptides [6, 42], etc.) were important in the regulating host energy metabolism and intestinal development and maintaining intestinal epithelial integrity etc. [43–46], as well as stimulating and regulating the maturation and differentiation of the host immune system [10].

Hence, the characteristics of these common predominant bacteria were universal in different mammals gastrointestinal tract, and which was the result of mutual adaptation and domestication between host and intestinal flora [47, 48], and reflected the consistency of co-evolutionary between the host and the intestinal flora in some degree [49].

### The relationship between different inductive sites and significant differential bacteria

At the genus level, there were significant differences in the relative abundance of the bacteria colonized ALNA and ileal PPs, which were mainly caused by Fibrobacteres in ALNA and *Clostridium (SMB53)* and *Escherichia* in ileal PPs. It was mainly related to the following reasons. First, the effects from the pH, bile salts and oxygen content, for example, the pH is about 5.55 (acidic) in the camel’s abomasum, but it is about 7.73 (alkaline) in the ileum [50]. The oxygen content is lower in the ileum than in the abomasum usually. The gram-negative bacteria have stronger tolerance of bile salts than gram-positive bacteria [51]. And in the gastrointestinal tract, the ileum is an important site for bile acids resorption, but the abomasum is not. Second, the difference from the component’s ratio of chyme from grazing plants etc., with the chyme advances backward in the gastrointestinal tract, the nutrients are gradually digested and absorbed, so the composition ratio of chyme are different, though they are all mainly from plants cellulose and their influences on the immunity were not clear. Third, there were differences in the mucus. In the abomasum, the average thickness of the mucus was about 700 μm, and mainly constituted by the MUC6 and MUC5AC, which were secreted by the mucous cells and epithelial cells, respectively. However, in the ileum, the average mucous thickness was about 150–300 μm, and were formed by the MUC2 [52]. Therefore, at the physiological conditions, the differences of them (such as pH, bile salts, the oxygen content, component’s ratio of chyme and the mucus) were the main factors that affect the bacteria at the genus level.

As well, the main differential bacteria could impact on the physiological functions. For example, the *F*. *succinogenes* was an important symbiotic bacterium that could degrade cellulose in the rumen of ruminants [36], and its metabolite succinic acid could successfully induce the ILC2 type immune response by the specific receptor [53]; and the *Bacteroides* could produce a weak agonistic activity of LPS (LPSBV), which could induce the semi-mature of the CD11c + bone marrow-derived dendritic cells, and this response did not induce the pro-inflammatory factors expression [39]. Moreover, some researches have shown that the lymph nodes located in the different intestinal segments perform “segmental” immune regulation on the intestine, for instance, the different immune responses were found in the different intestinal segments of mice attacked by pathogens, such as Salmonella [54]. And in our study, the abundance of the Fibrobacteres in the ALNA were significantly higher than in ileal PPs, the abundances of the *Clostridium* (*SMB53*) and *Escherichia* in ileal PPs were significantly higher in ALNA. Therefore, the colonization of the local differential bacteria makes the whole digestive tract mucosal immune homeostasis different among different intestinal segments.

In addition, some intestinal bacteria were not identified in this study, so the biological information hided in them needs further research and exploration. However, the results of the present study will lay a foundation for further research the role of microbial flora on gastrointestinal mucosal immunity.

## Supporting information

S1 Table Number of clean sequence reads and OTUs and the values of alpha diversity indices in each sample. (DOCX)

S2 Table The abundance of all the phyla identified in RMFR, LMFR and PPS. (DOCX)

S3 Table The abundance of all the genera identified in RMFR, LMFR and PPS. (DOCX)
