# Supplementary material for: Bacterial community analysis on the different mucosal immune inductive sites of gastrointestinal tract in Bactrian camels
Source: PLoS One. 2020 Oct 8;15(10):e0239987. doi: 10.1371/journal.pone.0239987 (PMC7544057; doi:10.1371/journal.pone.0239987)
Supplement: S1 Table — (DOCX) [file pone.0239987.s001.docx]

**S1 Table. Number of clean sequence reads and OTUs and the values of alpha diversity indices in each sample.**

| **Sample** | **Nseqs** | **NOTUs** | **alpha diversity** | | |
| --- | --- | --- | --- | --- | --- |
|  |  |  | **chao1** | **shannon** | **simpson** |
| RMFR-1 | 103740 | 689 | 728.9528 | 7.338285 | 0.984692 |
| RMFR-2 | 94438 | 719 | 764.9908 | 7.660821 | 0.988675 |
| RMFR-3 | 95258 | 576 | 615.1734 | 7.254083 | 0.985119 |
| RMFR-4 | 91052 | 594 | 645.6229 | 7.486825 | 0.989741 |
| RMFR-5 | 96798 | 592 | 621.6625 | 6.909129 | 0.976827 |
| RMFR-6 | 109730 | 615 | 692.9768 | 7.032829 | 0.98009 |
| LMFR-1 | 101008 | 715 | 765.0843 | 7.43154 | 0.985519 |
| LMFR-2 | 94370 | 719 | 758.0244 | 7.696769 | 0.989034 |
| LMFR-3 | 96906 | 595 | 645.5471 | 7.184701 | 0.983116 |
| LMFR-4 | 100676 | 614 | 658.3181 | 7.493264 | 0.988971 |
| LMFR-5 | 91754 | 576 | 635.6622 | 6.565686 | 0.964227 |
| LMFR-6 | 92756 | 570 | 616.8934 | 7.061598 | 0.981726 |
| PPs-1 | 95232 | 278 | 281.5192 | 7.009037 | 0.985296 |
| PPs-2 | 99354 | 391 | 395.298 | 4.794502 | 0.754843 |
| PPs-3 | 96618 | 433 | 450.8466 | 6.227164 | 0.944866 |
| PPs-4 | 95886 | 419 | 448.1375 | 6.291101 | 0.961663 |
| PPs-5 | 90350 | 182 | 193.183 | 3.416731 | 0.809543 |
| PPs-6 | 99374 | 450 | 477.0943 | 6.388893 | 0.958257 |

Note: Nseqs indicates the number of sequence clean reads; NOTUs indicates the number of OTUs
