# Supplementary material for: Bacterial community analysis on the different mucosal immune inductive sites of gastrointestinal tract in Bactrian camels
Source: PLoS One. 2020 Oct 8;15(10):e0239987. doi: 10.1371/journal.pone.0239987 (PMC7544057; doi:10.1371/journal.pone.0239987)
Supplement: S2 Table — (DOCX) [file pone.0239987.s002.docx]

**S2 Table. The abundance of all the phyla identified in RMFR, LMFR and PPS.**

| **Phylum** | **RMFR** | **LMFR** | **PPs** |
| --- | --- | --- | --- |
| Bacteroidetes | 32.98±5 | 34.43±8.64 | 23.87±11.9 |
| Firmicutes | 17.61±3.87 | 16.65±3.45 | 20.1±8.68 |
| Verrucomicrobia | 11.11±5.22 | 12.52±9.88 | 9.71±10.3 |
| Fibrobacteres | 4.5±3 | 3.83±1.57 | 0.26±0.44 |
| Proteobacteria | 4.33±3.66 | 4.86±3.38 | 18.03±19.89 |
| SR1 | 2.76±2.97 | 2.69±2.97 | 0.46±0.95 |
| Lentisphaerae | 2.07±0.88 | 1.81±0.87 | 3.45±2.91 |
| Elusimicrobia | 1.57±2.52 | 1.37±2 | 0.16±0.19 |
| Spirochaetes | 1.42±0.5 | 1.49±0.6 | 0.57±0.38 |
| Tenericutes | 1.17±0.69 | 1.43±1.05 | 0.63±0.48 |
| Cyanobacteria | 0.39±0.18 | 0.33±0.07 | 1.96±1.68 |
| TM7 | 0.18±0.13 | 0.24±0.15 | 0.12±0.17 |
| Synergistetes | 0.09±0.05 | 0.12±0.11 | 0±0 |
| OD1 | 0.01±0.01 | 0.01±0.01 | 0±0 |
| Actinobacteria | 0±0.01 | 0±0 | 0.01±0.02 |
| Chloroflexi | 0±0 | 0±0 | 0.03±0.04 |
| Deferribacteres | 0±0 | 0±0 | 0.04±0.06 |
| Fusobacteria | 0±0 | 0±0 | 3.87±9.49 |
| Others | 19.81±6.27 | 18.23±3.3 | 16.7±7.26 |

Note: the data are expressed as mean ± SD.
