# Supplementary material for: Bacterial community analysis on the different mucosal immune inductive sites of gastrointestinal tract in Bactrian camels
Source: PLoS One. 2020 Oct 8;15(10):e0239987. doi: 10.1371/journal.pone.0239987 (PMC7544057; doi:10.1371/journal.pone.0239987)
Supplement: S3 Table — (DOCX) [file pone.0239987.s003.docx]

**S3 Table. The abundance of all the genera identified in RMFR, LMFR and PPS.**

| Genus | RMFR | LMFR | PPs |
| --- | --- | --- | --- |
| Prevotella | 4.6729±3.2820 | 5.6353±5.3323 | 3.0921±4.9395 |
| Fibrobacter | 4.4928±3.0012 | 3.8303±1.5696 | 0.2647±0.4377 |
| RFN20 | 1.9469±1.2619 | 1.9338±0.9468 | 0.2074±0.4455 |
| Campylobacter | 1.8014±2.6950 | 2.4891±2.8715 | 0±0 |
| Succiniclasticum | 1.7562±0.7227 | 1.8259±0.8221 | 0.5811±1.2575 |
| BF311 | 1.2178±0.5594 | 1.2618±0.6140 | 0.2553±0.5159 |
| Elusimicrobium | 1.1562±2.6907 | 0.9586±2.1731 | 0.0371±0.0745 |
| Treponema | 0.9470±0.4098 | 0.9806±0.4361 | 0.4416±0.3407 |
| CF231 | 0.6411±0.2073 | 0.6328±0.2523 | 0.3674±0.2768 |
| Clostridium | 0.6171±0.5363 | 0.6103±0.4524 | 1.8244±2.2710 |
| Ruminococcus | 0.4398±0.1356 | 0.5090±0.2415 | 0.1256±0.1721 |
| Anaeroplasma | 0.4126±0.7400 | 0.6257±1.0570 | 0.0480±0.0538 |
| Butyrivibrio | 0.3652±0.2521 | 0.3751±0.2053 | 0.1536±0.2571 |
| YRC22 | 0.2477±0.3501 | 0.3195±0.4490 | 0.1896±0.4645 |
| Desulfovibrio | 0.1125±0.0527 | 0.0981±0.0524 | 0.0019±0.0047 |
| Moryella | 0.1112±0.1296 | 0.0954±0.1165 | 0.0374±0.0890 |
| Sphaerochaeta | 0.0946±0.0604 | 0.1088±0.0476 | 0.0536±0.1168 |
| Pyramidobacter | 0.0930±0.0529 | 0.1162±0.1089 | 0±0 |
| Oscillospira | 0.0673±0.0428 | 0.0706±0.0519 | 0.2894±0.2949 |
| Pseudobutyrivibrio | 0.0610±0.0568 | 0.0782±0.0605 | 0.0467±0.0490 |
| L7A_E11 | 0.0515±0.0268 | 0.0385±0.0236 | 0.0045±0.0110 |
| Succinivibrio | 0.0420±0.0586 | 0.0424±0.0225 | 0.0064±0.0157 |
| Coprococcus | 0.0210±0.0272 | 0.0195±0.0236 | 0.0068±0.0090 |
| Flexispira | 0.0178±0.0131 | 0.0158±0.0188 | 0±0 |
| Moraxella | 0.0171±0.0261 | 0.0101±0.0092 | 0±0 |
| Anaerostipes | 0.0164±0.0136 | 0.0176±0.0193 | 0.0053±0.0130 |
| Ruminobacter | 0.0142±0.0175 | 0.01601±0.0370 | 0±0 |
| Oxalobacter | 0.0138±0.0174 | 0.0130±0.0121 | 0±0 |
| Roseburia | 0.0123±0.0115 | 0.0041±0.0055 | 0.0890±0.1014 |
| Streptococcus | 0.0120±0.0250 | 0.0048±0.0062 | 0.0017±0.0027 |
| Bulleidia | 0.0099±0.0082 | 0.0184±0.0234 | 0.0049±0.0080 |
| Escherichia | 0.0092±0.0201 | 0.0064±0.0080 | 6.8277±13.7154 |
| Paludibacter | 0.0067±0.0078 | 0.0052±0.0056 | 0.1745±0.2054 |
| Anaerofustis | 0.0066±0.0023 | 0.0096±0.0094 | 0.0016±0.0039 |
| p-75-a5 | 0.0060±0.0102 | 0.0119±0.0117 | 0.0173±0.0290 |
| Lachnospira | 0.0051±0.0068 | 0.0129±0.0224 | 0±0 |
| Bacteroides | 0.00402±0.0065 | 0±0 | 0.6849±0.8931 |
| Blautia | 0.0039±0.0073 | 0.0007±0.0018 | 0.0625±0.0562 |
| 5-7N15 | 0.0038±0.0073 | 0±0 | 5.7500±6.2162 |
| Lactobacillus | 0.0024±0.0027 | 0.0016±0.0025 | 0.0111±0.0230 |
| Anaerovibrio | 0.0017±0.0043 | 0±0 | 0±0 |
| Akkermansia | 0.0016±0.00244 | 0±0 | 1.8732±2.1021 |
| SHD-231 | 0.0015±0.0024 | 0.0008±0.0021 | 0.0292±0.0384 |
| Mogibacterium | 0.0015±0.0037 | 0±0 | 0±0 |
| SMB53 | 0.0009±0.0021 | 0.0008±0.0021 | 1.9687±2.8646 |
| Neisseria | 0.0008±0.0020 | 0.0010±0.0025 | 0.0227±0.0533 |
| [Clostridium] | 0±0 | 0±0 | 0.0050±0.0063 |
| Alistipes | 0±0 | 0±0 | 0.0018±0.0043 |
| Butyricicoccus | 0±0 | 0±0 | 0.0077±0.0121 |
| Cetobacterium | 0±0 | 0±0 | 3.8702±9.4800 |
| Coprobacillus | 0±0 | 0±0 | 0.0427±0.0472 |
| Dorea | 0±0 | 0±0 | 0.0789±0.0840 |
| Epulopiscium | 0±0 | 0±0 | 1.2722±3.1042 |
| gut | 0±0 | 0±0 | 0.0275±0.0502 |
| Mucispirillum | 0±0 | 0±0 | 0.0428±0.0601 |
| Odoribacter | 0±0 | 0±0 | 0.0505±0.0699 |
| Parabacteroides | 0±0 | 0±0 | 0.0401±0.0469 |
| Phascolarctobacterium | 0±0 | 0±0 | 0.2010±0.2170 |
| Propionibacterium | 0±0 | 0±0 | 0.0086±0.0211 |
| Pseudomonas | 0±0 | 0±0 | 0.0796±0.195 |
| Psychrobacter | 0±0 | 0±0 | 0.0647±0.1585 |
| Rahnella | 0±0 | 0±0 | 0.0070±0.0173 |
| rc4-4 | 0±0 | 0±0 | 0.1252±0.1290 |
| Rhodanobacter | 0±0 | 0±0 | 0.0219±0.0536 |
| Shigella | 0±0 | 0±0 | 0.0103±0.0130 |
| Sutterella | 0±0 | 0±0 | 0.0368±0.0484 |
| Turicibacter | 0±0 | 0±0 | 0.7164±1.0710 |
| Victivallis | 0±0 | 0±0 | 0.0031±0.0077 |
| Others | 78.4620±2.2742 | 77.1937±6.7050 | 67.7269±24.3675 |

Note: the data are expressed as mean ± SD.
